# *Diospyros abyssinica* (Hiern) F.White (Ebenaceae), a new distributional record for Rwanda

**DOI:** 10.3897/BDJ.14.e189867

**Published:** 2026-06-10

**Authors:** Michael B Thomas, Elias Bizuru, Concorde Nsengumuremyi, Gaspard Munyaneza

**Affiliations:** 1 Affiliate Researcher, National Herbarium, Center of Excellence in Biodiversity & Natural Resource Management, University of Rwanda, Huye, Rwanda Affiliate Researcher, National Herbarium, Center of Excellence in Biodiversity & Natural Resource Management, University of Rwanda Huye Rwanda; 2 Rwanda Forestry Authority, Kigali, Rwanda Rwanda Forestry Authority Kigali Rwanda; 3 University of Rwanda, Kigali, Rwanda University of Rwanda Kigali Rwanda

**Keywords:** biodiversity, botany, checklist, conservation, distribution, Eastern Province, ex situ, flora, herbaria, herbarium, inventory, in situ, plant, Rwanda, species discovery, specimens, taxonomy

## Abstract

**Background:**

The native vascular flora of Rwanda is currently the subject of ongoing study aimed at producing a comprehensive and up-to-date national checklist. The discovery of *Diospyros abyssinica* forms part of the Flora of Akagera National Park project, a focused initiative designed to document and synthesise the vascular plant diversity of this protected area in Rwanda’s Eastern Province. The ultimate objective of this project is to generate a complete and authoritative checklist of the Park’s vascular plant species, incorporating current taxonomic treatments, verified distributional data and conservation status assessments. The checklist is being developed through the integration of systematic field observations, the assembly and analysis of a comprehensive database of herbarium specimens and a critical review of relevant published and unpublished botanical literature.

**New information:**

A new country-level record of a native vascular plant species previously unrecorded in Rwanda emerged during 2023 fieldwork inventory in the Eastern Province. *Diospyros abyssinica* is reported as new to the Province, where it is represented in a relict population of only 15 individuals in less than 20 hectares. Due to the species having many uses, it has been prioritised as a species for indigenous tree planting programmes.

## Introduction

Considerable progress has been made in the exploration of the flora of Rwanda since the early botanical explorations of the country's rainforests in 1898 by Richard Kandt, who is best known for his role in identifying the source of the White Nile. Since that time, Rwanda's flora has been systematically collected and studied. These efforts have culminated in the documentation of species identified through the compilation of Belgian botanists Georges Troupin's four-volume Flore du Rwanda (Troupin 1978, Troupin 1983, Troupin 1985, Troupin 1988). More recently, Sosef et al. (2017) recorded 1,883 observed vascular plant species for Rwanda and estimated the country’s flora to comprise approximately 2,608 species. At a broader regional scale, the *Flore d’Afrique Centrale* project has estimated the combined flora of the Democratic Republic of the Congo, Rwanda and Burundi to comprise approximately 11,000 vascular plant species. Decades of additional botanical exploration and forest inventories have culminated into the development of several other seminal publications and field guides including Étude phytocenologique du Parcnational de l'Akagera et du Rwanda oriental (Troupin 1966), Plantes ligneuses du Parc National de L'Akagera (Troupin and Girardin 1975), Plants of Nyungwe National Park (Fischer and Killmann 2008), Les Plantes ligneuses du Rwanda (Bloesch et al. 2009) and The Orchids of Rwanda (Fischer et al. 2010). Despite this intensive research and botanical exploration, numerous new species continue to be discovered in Rwanda suggesting that many additional taxa remain to be discovered (Fischer et al. 2003, Fischer et al. 2013, Fischer and Killmann 2015, Fischer 2017, Fischer and Ackermann 2019, Fischer et al. 2022, Fischer et al. 2023).

Today, the National Herbarium of Rwanda (NHR) functions as the country’s principal centre for botanical research and the primary repository for more than 22,000 plant specimens frequently cited in literature. Despite this, the majority of Rwandan herbarium material — particularly type specimens — remains housed outside the country in Northern Hemisphere herbaria, reflecting the colonial legacy of African plant taxonomy (Park et al. 2023). The concentration of historical African plant collections in the Northern Hemisphere, however, should not be regarded as an unchangeable reality, but rather as a structural imbalance that can be addressed and corrected (Figueirido and Smith 2010).

Recently, efforts to digitise plant specimens deposited outside of Rwandan have been prioritised, with many specimens now being delivered digitally through virtual herbaria and online data portals, such as the Meise Botanic Garden Herbarium (Meise Botanical Garden 2026) and the African Plants Portal (African Plants Portal 2026). This new data portal is a recent large-scale effort designed to establish an important biodiversity data resource, supported by the U.S. National Science Foundation, which is digitising more than 1.1 million herbarium specimens and associated data records from across tropical Africa, housed in 21 U.S. herbaria.

Rwanda developed its first National Biodiversity Strategy and Action Plan (NBSAP) in 2003 after identification of major threats to biodiversity conservation (REMA 2016). Much of the Rwanda's biodiversity is preserved today within its four national parks including Nyungwe National Park (Fischer et al. 2024), Volcanoes National Park, Giswati-Mukura National Park and Akagera National Park. This species occurrence discovery is a component of the project 'Flora of Akagera National Park', which ultimately aims at the complete checklist including updated taxonomy, distribution and status of vascular plant species of Akagera National Park in the Eastern Province (Thomas and CoEB 2025).

Akagera National Park is Central Africa's largest protected wetland and the last remaining refuge for savannah-adapted species in Rwanda. The Akagera Complex Wetland comprises a transboundary network of wetlands lying between 1°18′–02°11′S and 30°33′–31°01′E, where it forms the borderland between Rwanda and Tanzania (Ndayisaba 2017). The wetland is traversed by the Akagera River which flows northwards into Lake Victoria. It marks the periphery of the Akagera National Park and is home to various wild animals, especially hippos, buffaloes, giraffes, impalas and sitatunga and constitutes an important hydrological reservoir that provides water to animals. Under the natural reserve protection laws in Rwanda, human activities are limited in this area. However, there are still some anthropogenic activities that continue to exert pressure on these swamps, notably agriculture, cattle grazing, production of loam bricks and the cutting of plants for animal feeding and construction purposes, especially at swamp edges (Fischer 2011).

## Materials and methods

**Figure 1.**
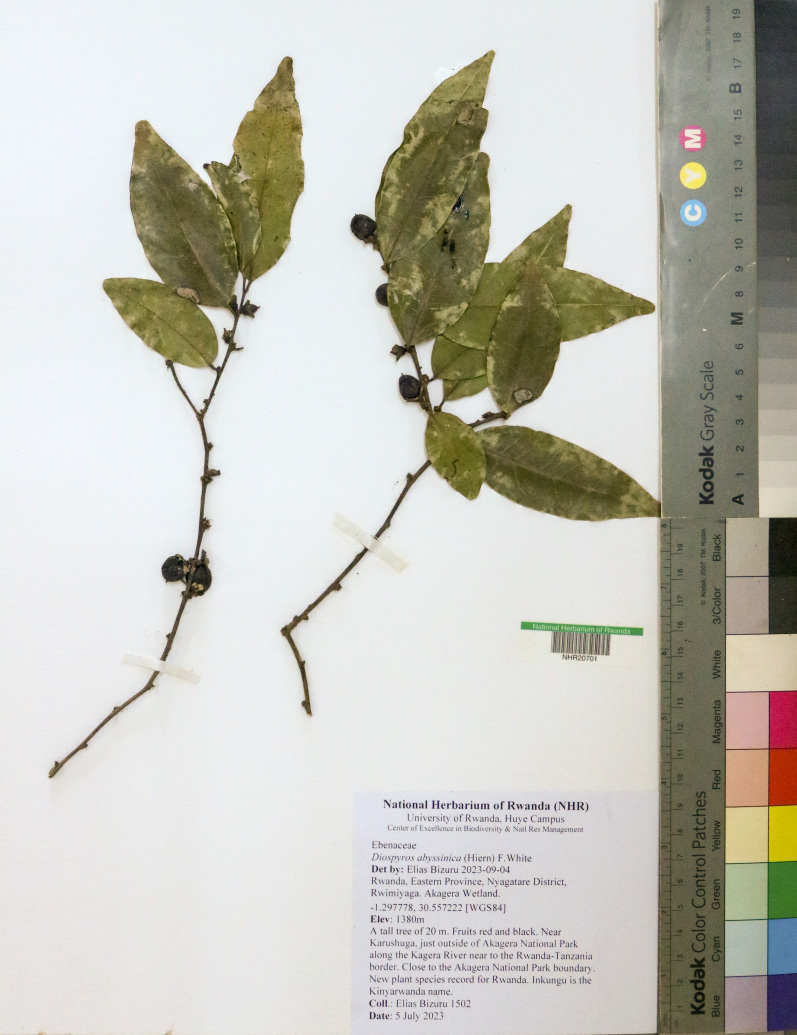
*Diospyros abyssinica* herbarium voucher specimen, #NHR20071.

The new information on this native vascular plant species was gathered by Elias Bizuru in the Eastern Province region during survey fieldwork on the COMBIO project in September 2023. A specimen was collected, prepared and deposited into the National Herbarium of Rwanda (Fig. 1). During this fieldwork, plant surveys were conducted near the village of Karushuga located along the western edge of the of the Kagera River. In compilation and verification with the Updated Checklist of Vascular Plants of Akagera National Park (in prep.), all available herbarium collections at NHR, EA and BR, herbarium codes according to Thiers (2025), were reviewed for occurrences in this region. The taxon was treated according to the methodology and data structure similar to that employed by the Flora of Zambesica (White 1983). The biogeographical range information for assessing distribution in the neighbouring countries was largely derived from POWO (2025) and various taxonomic authorities (White 1983, Bamps 1987, Beentje 1994, Dharani 2019, BGCI 2025, Dressler et al. 2025, iNaturalist 2025, POWO 2025, WFO 2025, Anonymous 2026, EOL 2026).

## Data resources

The herbarium specimen data were accessioned into the Rwanda Biodiversity Specimen Portal (Thomas and CoEB 2025) and the occurrence record was uploaded into the Rwanda Biodiversity Information System (RBIS). The individual herbarium specimen (NHR20701) was deposited and digitally imaged at the National Herbarium of Rwanda. The new record was georeferenced and made available through GBIF.

## Taxon treatments

### Diospyros abyssinica

(Hiern) F.White

B01CC6F1-E80E-5A3E-8871-13856600C66A

https://portal.boldsystems.org/result?query=%22Diospyros%20abyssinica%22[tax]

https://www.biodiversitylibrary.org/search?searchTerm=Diospyros%20abyssinica#/names

https://www.checklistbank.org/dataset/313531/taxon/36DSD

https://eol.org/pages/5238326

https://www.gbif.org/species/6056984

https://ipni.org/n/322036-1

https://powo.science.kew.org/taxon/urn:lsid:ipni.org:names:322036

https://www.worldfloraonline.org/taxon/wfo-0000648453

*Diospyros abyssinica* (Hiern) F. White. This species is accepted by POWO. Published in Bull. Jard. Bot. État Bruxelles 26: 241 (1956) (Conservatoire et Jardin botaniques de la Ville de Genève and South African National Biodiversity Institute 2025).
**Basionym**: *Maba abyssinica* Hiern.
**Homotypic Synonyms**: Diospyros abyssinica (Hiern) F.White subsp. abyssinica (1956); *Mabaabyssinica* Hiern (1873).
**Heterotypic** *Diospyros welwitschii* Hiern (1898); *Maba mualala* Welw. ex Hiern(1873); *Diospyros ubanghensis* A. Chev. (1911); *Maba ubanghensis* A. Chev. (1913); *Maba warneckei* Gürke (1911).

#### Materials

- **Type status:** Other material. **Occurrence:** catalogNumber: NHR20701; recordNumber: 1502; recordedBy: Elias Bizuru; reproductiveCondition: fruit-bearing; establishmentMeans: indigenous; occurrenceStatus: present; occurrenceID: 2A2E1D39-790B-535C-8E1A-1F8C8DFA167D; **Taxon:** originalNameUsageID: https://www.gbif.org/species/6056984; namePublishedInID: Bull. Jard. Bot. État Bruxelles 26: 241 (1956); scientificName: Diospyros abyssinica; kingdom: Plantae; order: Ebanales; family: Ebenaceae; genus: Diospyros; specificEpithet: abyssinica; taxonRank: species; scientificNameAuthorship: (Hiern) F.White; vernacularName: Inkunga; nomenclaturalCode: ICBN; taxonomicStatus: accepted; **Location:** higherGeography: Central Africa; continent: Africa; country: Rwanda; countryCode: RW; stateProvince: Eastern Province; county: Nyagatare; municipality: Rwimiyaga; locality: near Karushuga, just outside of Akagera National Park along the Kagera River near to the Rwanda-Tanzania border; verbatimLocality: just outside of Akagera National Park along the Kagera River near to the Rwanda-Tanzania border; verbatimElevation: 1380 m; verbatimCoordinates: decimal degrees; verbatimLatitude: -1.30184; verbatimLongitude: 30.55582; decimalLatitude: -1.30184; decimalLongitude: 30.55582; geodeticDatum: WGS84; coordinateUncertaintyInMeters: 10; georeferencedBy: Michael B. Thomas; georeferenceProtocol: "Guide to Best Practices for Georeferencing" (Chapman and Wieczorek, eds. 2006); georeferenceVerificationStatus: verified by collector and verified by Curator; **Identification:** identifiedBy: Elias Bizuru; dateIdentified: 2023-09-04; identificationReferences: Flora of Tropical East Africa; **Record Level:** type: PhysicalObject; language: en; rights: Content licensed under Attribution-NonCommercial-ShareAlike 4.0 International; rightsHolder: The Regents of the University of Rwanda; accessRights: public domain; bibliographicCitation: not-for-profit use only; institutionID: UR; collectionID: NHR; institutionCode: CoEB; collectionCode: NHR; ownerInstitutionCode: UR; basisOfRecord: PreservedSpecimen; source: https://rwandabiodiversity.net/collections/individual/index.php?occid=1586&clid=0

#### Description

*Diospyros abyssinica* is a large and tall tree of 30-35 m in height with a dense branch canopy; bole straight and cylindical with a DBH that varies between 23 and 47 cm for mature trees; bark dark grey to blackish-brown, rough and fissured with longitidunal cracks in older trees, but relatively smoother in younger individuals (Fig. 8), bark thickness increases with age to provide a protection against fire; wood very hard, white with blackish streaks in the heartwood visible inside both the bole and branches (Fig. 9). Leaves glabrous, coriaceous, green at younger age and becoming grey-green when old lanceolate-elliptic or elliptic, 7.4 - 13,4 cm long and 3.8-4.7 cm wide for mature leaves, sub-obtuse to acuminate at the apex or distinctly acuminate at the apex, leaves base obtuse to distinctly cuneate; principal nerve more conspicuous beneath the blade, lateral nerves 8-12 in number visible on both surfaces of the blade and coated with waxy layer to reduce evapotranspiration (Figs 10, 11, 12); petiole 5.2-6.3 mm long; fruits are red coloured or black at maturity, glabrous, subglobose or ellipsoid-subglobose; calyx persistent and patelliform. Seeds black, 1 globose to subellispoid. Female flowers (1-)3-5(-8) in axillary orramuligerous fascicles; pedicels 2 mm long, fulvous-setulose; calyx cyathiform, 6 mm long, glabrous outside, finely strigulose towards the base inside, divided almost to the base; calyx-lobes 3-4, strongly imbricate, ± round, 6 mm long and wide, sometimes apiculate; corolla similar to that of male, slightly shorter than the calyx; staminodes 3-4, filiform, 2 mm long, glabrous, attached to the corolla-throat and alternating with the lobes, exserted; ovary conical, 4 mm long, 2 mm wide, glabrous, gradually merging into the short undivided style; locules 6, uniovulate; stigmatic lobes 3, ± 1 mm long, ascending. Male flowers peach-scented, 10-18 in contracted cymes, axillary or borne on branchlets; peduncle 1 mm long; pedicels 1 mm long, fulvoussetulose; calyx shallowly cyathiform, 2 mm long; calyx-lobes 3-4, broadly triangular, glabrous outside, except for a few minute marginal hairs, glabrous inside; corolla cream, subrotate, 5-6 mm long, glabrous; corolla-tube 1.5 mm long; corolla-lobes 3-4, broadly elliptic, 4-5 mm long, 3 mm wide, obtuse at the apex; stamens 10-15, 2-4 mm long; anthers lanceolate-apiculate, sparsely setulose towards the apex; rudimentary ovary 1 mm long, glabrous, sometimes absent. Fruit red or black at maturity, ellipsoid to subglobose, 9.2-10.6 mm long, 7.2-8.1 mm wide, glabrous, with persistent style; calyx persistent after fruits maturation, becoming patelliform, 3.8-4.6 mm long (Figs 3, 4). Seeds 1 per fruit, subellipsoid, yellow to grey black, 3.7 large and 5.7 mm long with a longitudinal back line (Fig. 13); endosperm smooth.

#### Diagnosis

**Figure 2.**
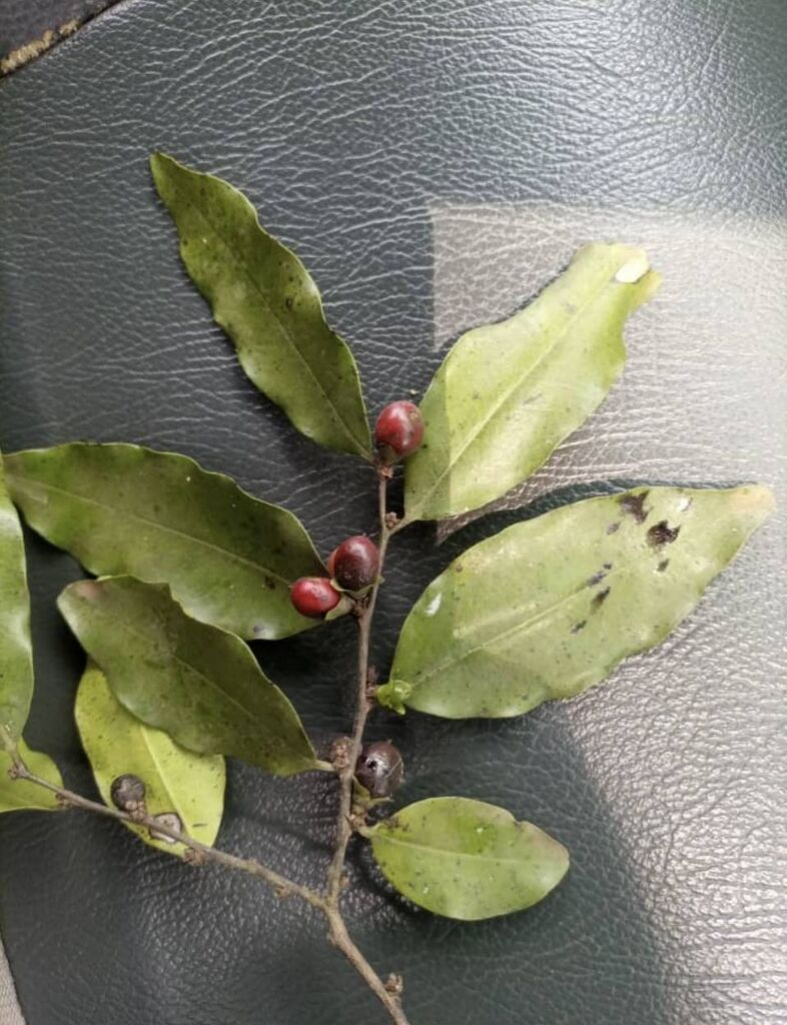
*Diospyros abyssinica*, lanceolate-elliptic leaves, rounded to cuneate at the base, subcoriaceous, glabrescent on lower surface; ripening small red-black fruit in cup-shaped remainder of calyx.

**Figure 3.**
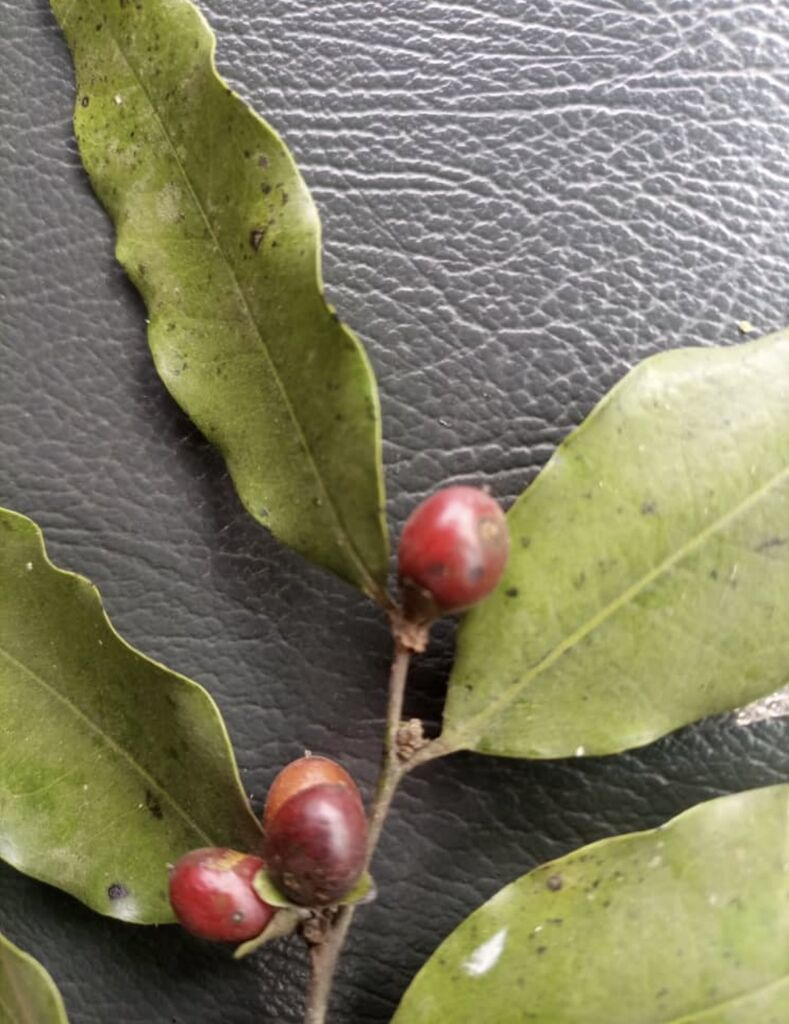
*Diospyros abyssinica*, closeup of ripening single or sometimes 2-seeded fruit.

**Figure 4.**
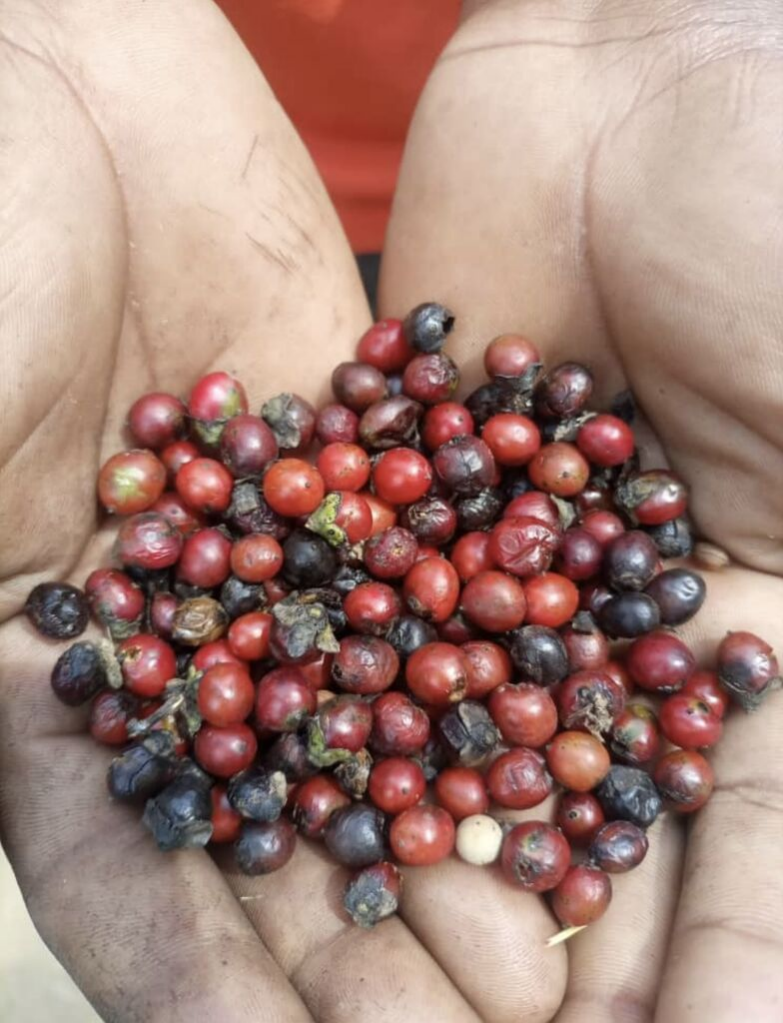
*Diospyros abyssinica*, red-black mature fruits.

*Diospyros abyssinica* differs significantly from *D. gabunensis*, the only other native to Rwanda, morphologically by the leaf and fruit size, fruit colour and seed numbers. *Diospyros abyssinica*, has an orange or red to yellow fruit, 0.8-1.4 cm long, 8-9 mm wide and the calyx is scarcely accrescent and contains only one black seed (very rarely 2), while *D. gabunensis* fruit is blackish or purplish, ca. 2.5(3) cm in diameter and the calyx accrescent with 8-10 seeds (Figs 2, 3, 4). In addition, *D. abyssinica* leaves are much smaller, measuring 7.4-11.6 cm long, 3.8-4.7 cm wide whereas those of *D. gabunensis* are ca. 18 x 6.5 cm.

#### Distribution

The broad native range of *Diospyros abyssinica* is Tropical or Sub-Saharan Africa. It is known to be native to: Angola, Benin, Burkina, Cameroon, Central African Republic, Chad, Eritrea, Ethiopia, Gabon, Ghana, Guinea, Ivory Coast, Kenya, Malawi, Mali, Mozambique, Nigeria, Sudan, Tanzania, Togo, Uganda, Zambia, Zaïre and Zimbabwe (EOL 2026, POWO 2025).

#### Ecology

It is a shrub or tree and occurs mainly in moist tropical and subtropical woodland and forest ecosystems. It occurs in coastal and highland forests at altitudes up to 2200 m (Dharani 2019).

#### Conservation

*Diospyros abyssinica* was most recently assessed for the **IUCN Red List of Threatened Species** in 2020 and is currently classified as Least Concern (Beentje et al. 2025). Despite its global status, the species is represented in Rwanda by fewer than 20 known individuals, all restricted to Karushuga, in an area adjacent to **Akagera National Park**. Immediate threats at this site include potential harvesting by local communities for timber and firewood.

*D. abyssinica* is known from six international ex-situ collections, including sites in Benin, Kenya and the United States, with additional seed material conserved at the **Millennium Seed Bank** (BGCI 2025). At present, the species is not formally represented in any ex-situ conservation collection within Rwanda.

**Figure 5.**
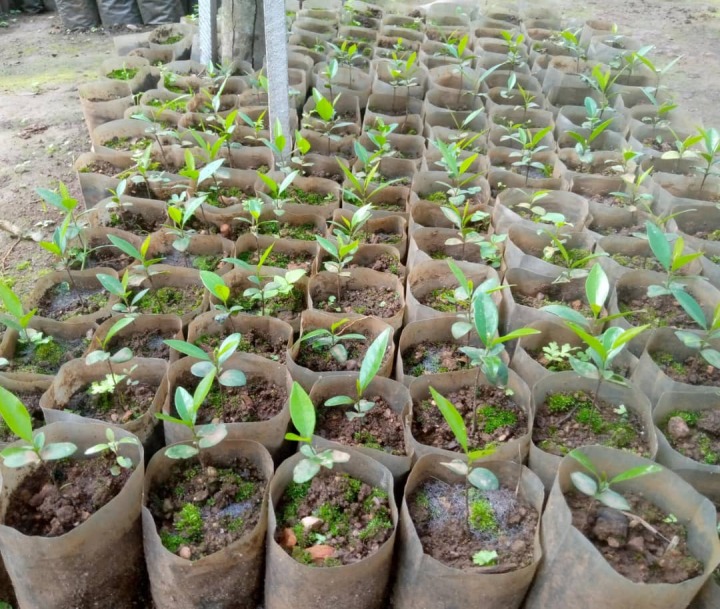
Three month old *D. abyssinica* seedlings growing for out-planting in a local nursery.

**Figure 6.**
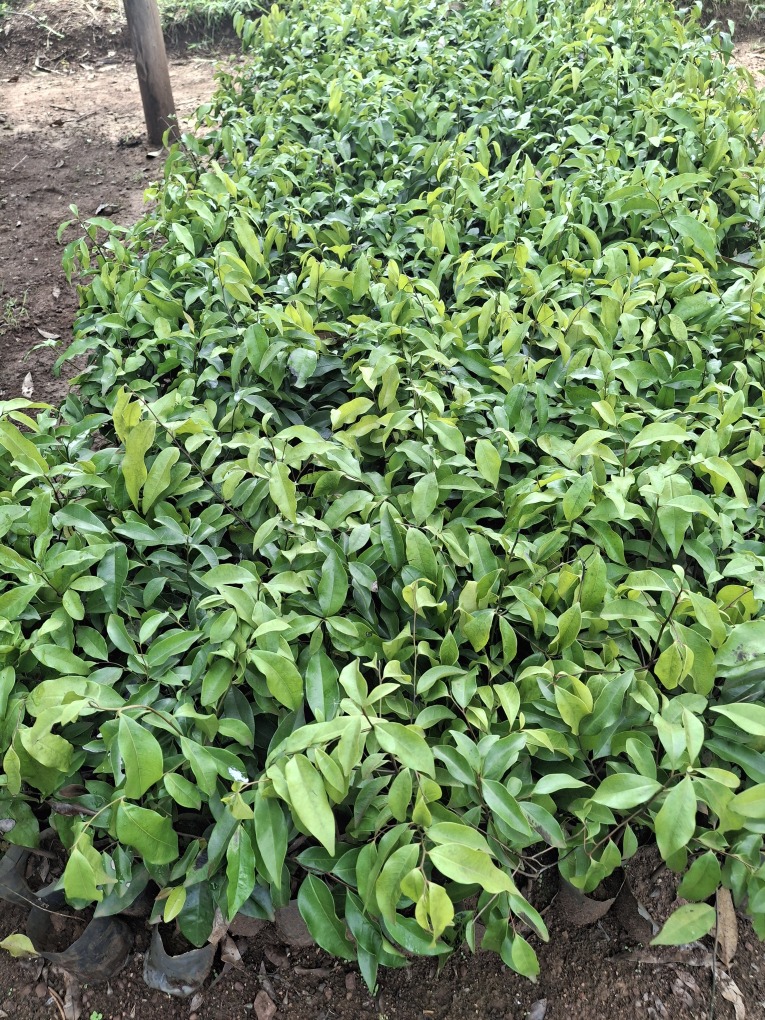
Seedlings of *D. abyssinica* after one year in a comunity nursery of Gahini/Kayonza, Eastern Province of Rwanda.

**Figure 7.**
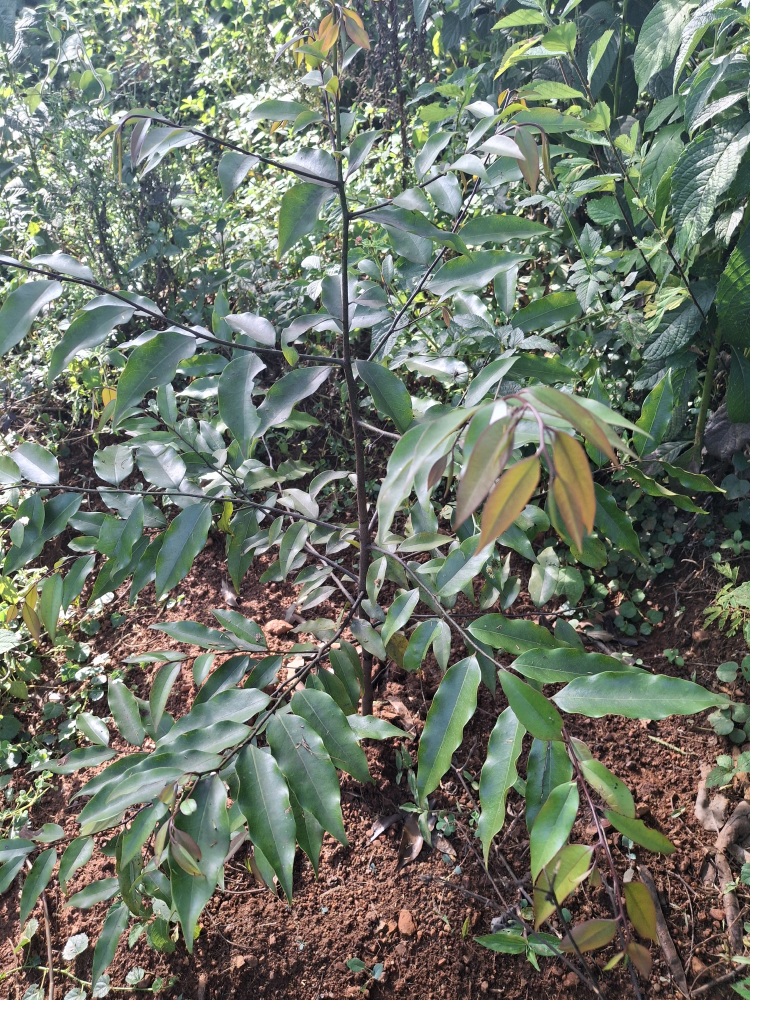
A one year *D. abyssinica* planted within the Gahini Community Biodiversity Sanctuary.

**Figure 8.**
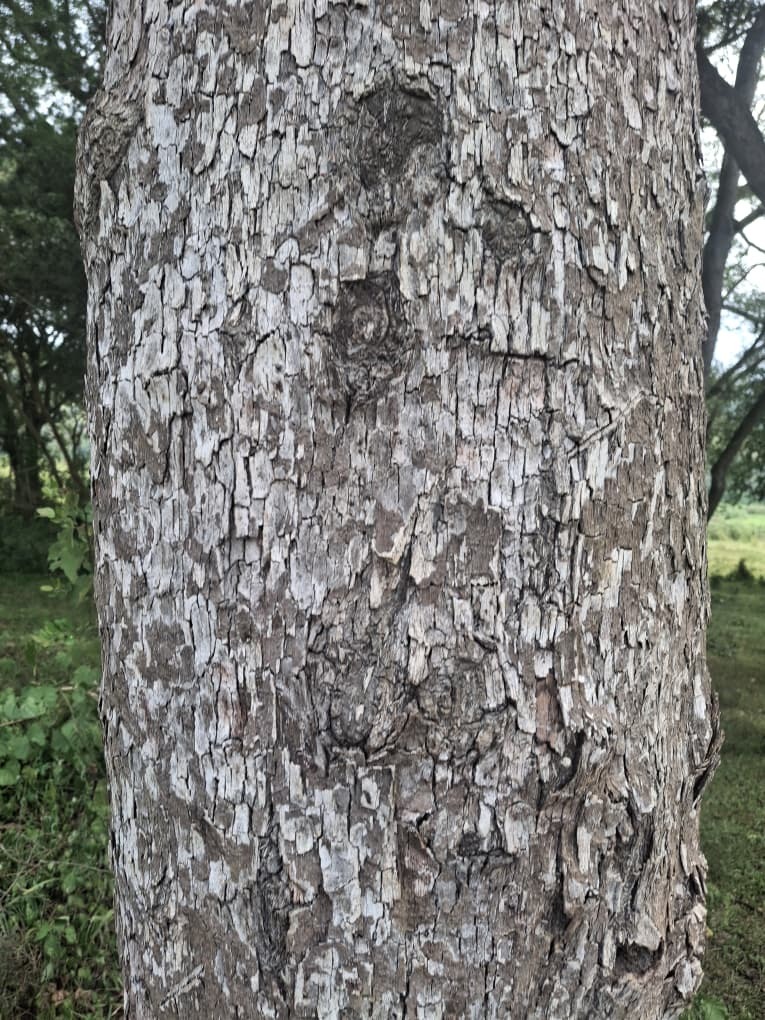
Bark of insitu specimen of *Diospyros abyssinica*.

**Figure 9.**
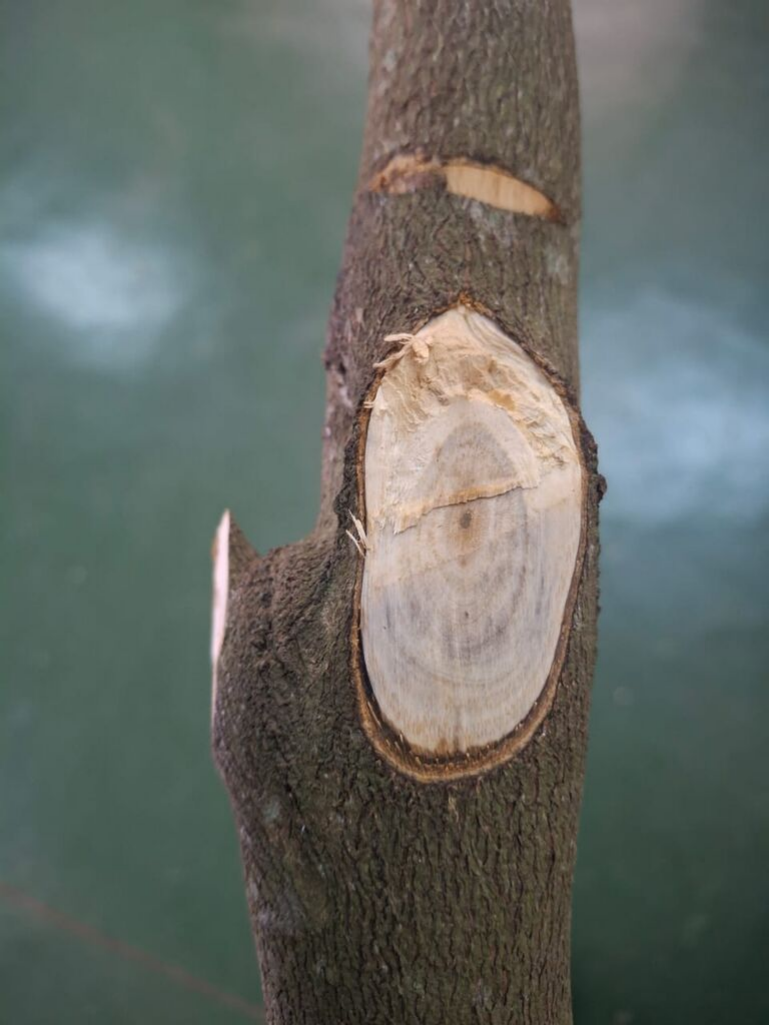
Blackish streaks of *Diospyros abyssinica* branch inner wood.

**Figure 10.**
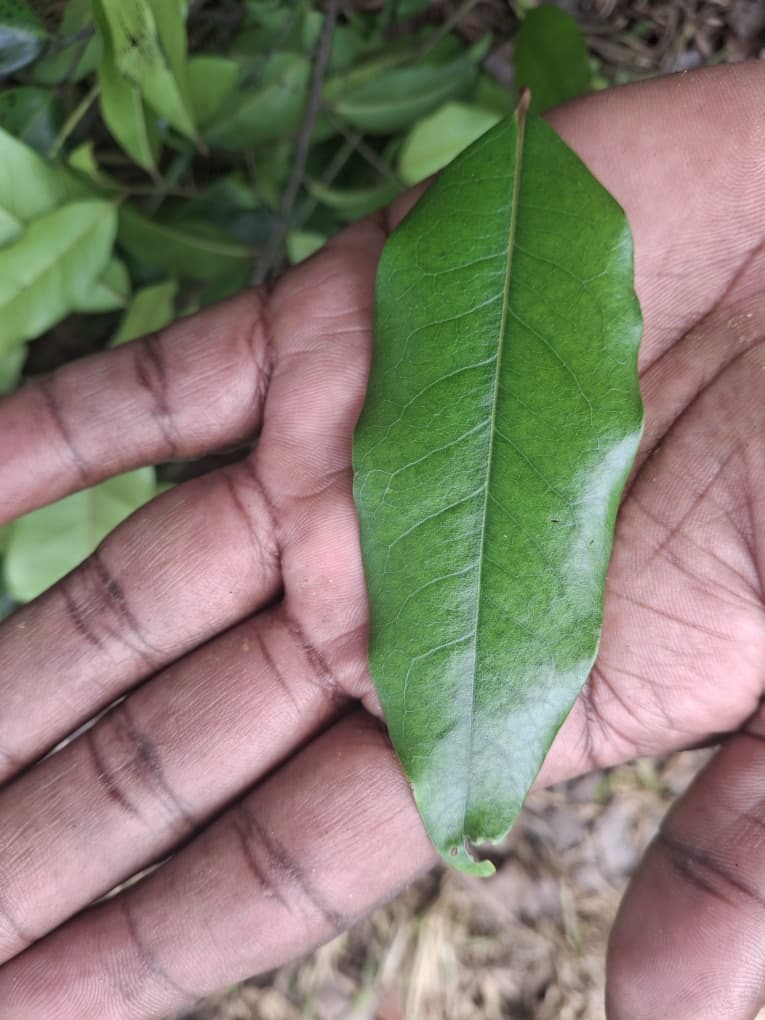
Fresh leaf of *Diospyros abyssinica*.

**Figure 11.**
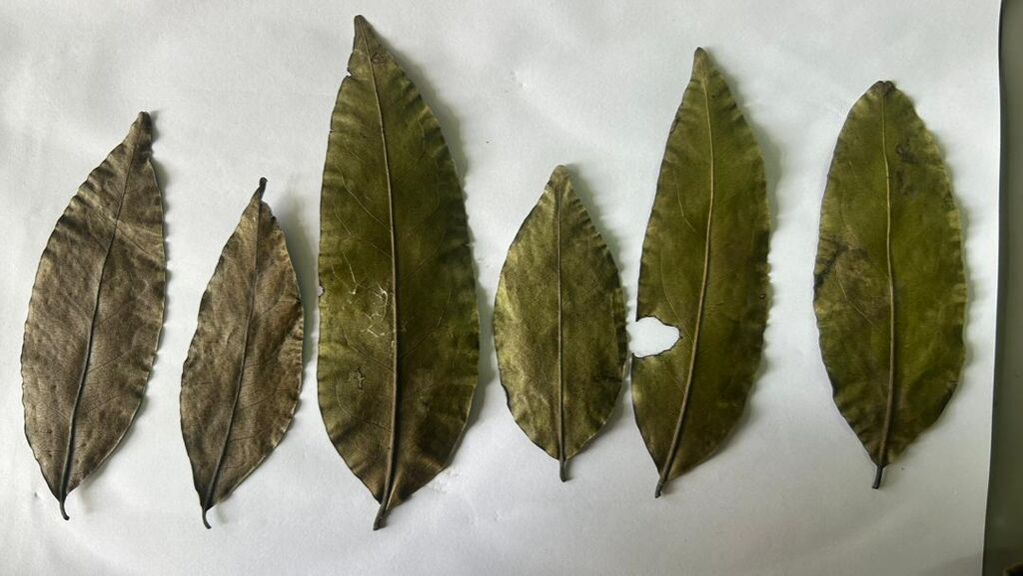
Various shapes of leaves of *D. abyssinica* (Abaxial view).

**Figure 12.**
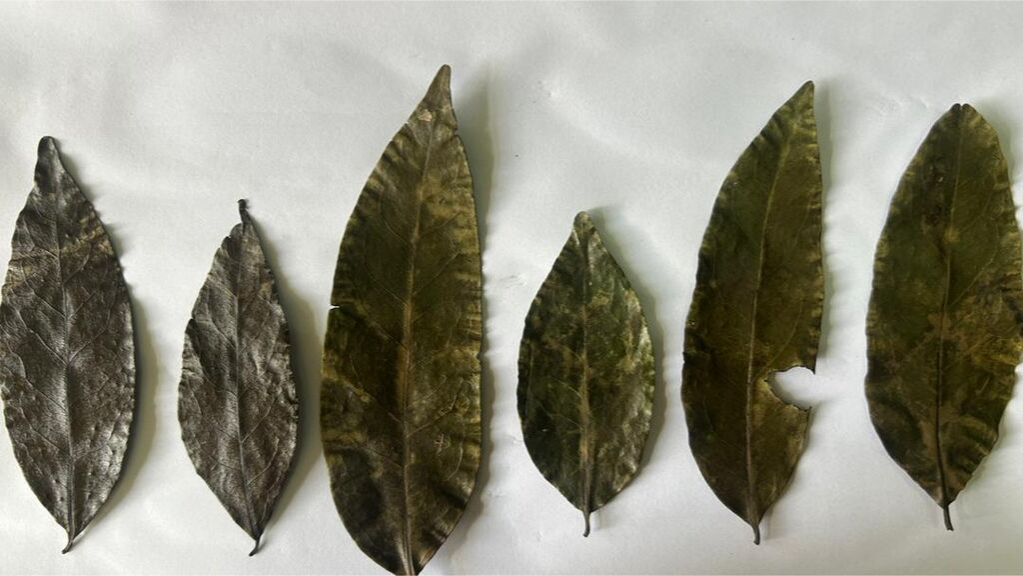
*D. abyssinica* leaves (Adaxial view).

**Figure 13.**
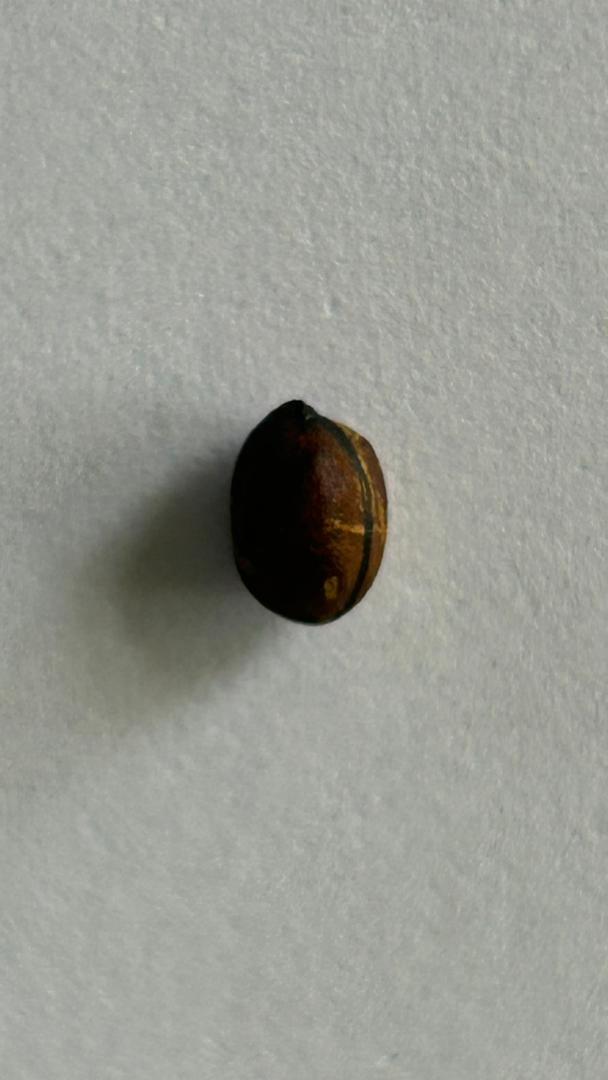
Seed of *Diospyros abyssinica*.

Between 2023 and 2026, nursery propagation efforts in Rwanda resulted in the production of nearly 6,000 seedlings, of which 2,843 trees were out-planted at Community Biodiversity Sanctuaries (CBS) in Kayonza and Gatsibo Districts, Eastern Province (Table 1; Figs 5, 6, 7). However, because these seedlings originated from a limited local gene pool, there is concern that the out-planted populations may possess reduced genetic diversity. The nearest previously documented populations occur in southern Uganda, approximately 100–150 km from Rwanda, while it is more widespread in Tanzania, particularly in the Kagera Region near Lake Victoria, approximately 300–350 km away.

#### Biology

The species is a shrub to tall forest tree, up to 30 m with a relatively sparse and shortly branched mushroom-shaped crown (Beentje 1994). Like other *Diospyros*, it is an evergreen species and dioecious, with male and female flowers borne on separate individuals. This reproductive biology has important implications for both propagation and conservation, in situ and ex situ.

As only female trees produce fruit and seed, successful natural regeneration depends on the presence of reproductively mature male trees within pollination distance. Fragmentation of forest habitats or selective removal of individuals can, therefore, reduce effective pollination and seed production, even where apparently healthy populations remain. In small or isolated populations, skewed sex ratios may further limit reproductive success and reduce genetic diversity over time.

For ex situ conservation and restoration programmes, dioecy requires careful planning to ensure that both male and female individuals are represented in living collections, seed orchards and re-introduction efforts. Propagation from seed alone does not guarantee balanced sex ratios, since the sex of seedlings is generally unknown until flowering. Vegetative propagation of known male and female trees may, therefore, be important for establishing breeding populations and maintaining long-term reproductive viability.

These considerations are particularly relevant for restoration and conservation initiatives involving fragmented tropical forest ecosystems, where maintaining functional population structure is essential for sustaining natural regeneration and long-term species persistence.

#### Taxon discussion

This taxon has not previously been reported for the flora of Rwanda, thus this paper representing its first verified record. Its occurrence extends the known distribution range of the species in Central Africa and contributes to completing the floristic knowledge of Rwanda. No earlier confirmed records exist in the national or regional literature.

#### Notes

We note that *Diospyros abyssinica* was inadvertently included in Fischer et al. (2024), based on a misinterpretation of published data. The species record was believed to be derived from Troupin (1982), who, in this short paper, describes only Acanthaceae, Melastomataceae and Sapotaceae and makes no reference to *D. abyssinica*. Troupin’s “Plantae africanae. IX (Acanthaceae, Melastomataceae, Sapotaceae)”, was cited to be published in Vol. 52, No. 3/4 (31 Dec 1982), pp. 163-165 — but the widely used, database-supported pagination for it is pp. 463–465, not pp 163-165. This is simply a typographical error. The correct place to review this species is Troupin’s Flore du Rwanda (Spermatophytes) (Troupin 1985, Vol. III), where Ebenaceae are treated and one can see there is no reference to *D. abyssinica*. In addition, Fischer et al. (2024) reports *Drosera pilosa* Exell & Laundon as being in Ebenaceae with *Diospyros gabunensis* Gürke in Ehretiaceae, which appears to be a row misalignment error. So we conclude this is the first documentation of this taxon as a new distribution record in Rwanda. We clarify here that the original attribution and taxon record belonging to Fischer et al. (2024) and the checklist inclusion should be regarded as an error.

#### Ethnobotany - Uses

The fruit is not known to be consumed by people in Rwanda. In other countries, fruits are reported to be eaten by birds and small mammals and are known to be an important component of the diet of both primates and fruit bats (Sumner and Mollon 2000). Besides the varied use of its hard and moderately heavy wood, the dark colour and high durability of other *Diospyros* species are lacking. *Diospyros abyssinica* is recorded to have medicinal properties in neighbouring countries ethnopharmacopaeia (Mallavadhani et al. 1998, Diallo et al. 2000). The wood of *Diospyros abyssinica* is used for heavy flooring, poles, interior trim, mine props, furniture, cabinet making, masts of dhows (traditional sailing vessel), agricultural implements, musical instruments, tool handles, ladders, toys, novelties, pestles, mortars, golf club heads, sticks, loom shuttles in weaving sisal cloth, carving and turnery (Obeng 2010). The wood is commonly used as firewood and for charcoal production. Various parts of the plants are reportedly used in traditional medicine (Ribeiro 2023). In Ghana, bark and roots are used by the Krobo people against various diseases (Obeng 2010). In Mali, leaf and roots decoctions are used to treat malaria and dysentery and to promote wound healing (Maiga et al. 2006, Odlo 2011). In Tanzania, a root decoction is taken to treat leprosy (Obeng 2010). Unfortunately, ethnobotanical data supporting its use in Rwanda as a traditional medicine is lacking.

## Discussion

Prior to this study, the Ebenaceae of Rwanda were thought to comprise just three confirmed taxa: *Diospyros gabunensis* Gürke, *Euclea divinorum* Hiern and E. racemosa subsp. schimperi (A.DC.) F.White. All taxa are of low conservation concern as they are widely distributed throughout Tropical Africa; they are listed as Least Concern (LC) on the IUCN Red List. In Rwanda, *D. abyssinca* is restricted to montane forests of Nyungwe National Park from 1,750–2,200 m, where it typically occurs in cold, moist microhabitats characteristic of the upper-montane zone (Troupin 1985, Fischer et al. 2024). The remaining two Euclea species are smaller shrubs or small trees, 2-8 m tall. *Euclea divinorum* occurs in wooded ravines, secondary forest regrowth, wooded swamps and wooded savannahs at 1,400–1,700 m elevation (Troupin 1985). E. racemosa subsp. schimperi occurs in gallery forests, xerophilous forests and thickets and wooded savannahs at 1,350–1,500 m elevation (Troupin 1985).

The confirmation of *Diospyros abyssinica* as occurring in Rwanda therefore adds an important fourth member to Ebenaceae and marks the first documented occurrence of this widespread Eastern and Central Africa taxon in the country. With this record, *Diospyros* is now represented by two taxa in Rwanda. The addition of *D. abyssinica* contributes to ongoing efforts to improve documentation of Rwanda’s flora and highlights the value of continued botanical surveys across a range of ecosystems. Further fieldwork in habitats that have historically received limited attention, including aquatic systems, ephemeral wetlands, upper montane forests and serpentine landscapes, may help improve understanding of the country’s plant diversity and distribution patterns.

## Supplementary Material

XML Treatment for Diospyros abyssinica

## Figures and Tables

**Table 1.** Number of D. abyssinica seedlings cultivated and out-planted.

**Community Biodiversity Sanctuary (CBS)**	**Number of seedlings Propagated**	**Number of Seedlings Planted into CBS**	**Number of seedlings Donated**	**Out-planted**	**Balance in Nursery**
Gahini/Kayonza	2,646	898	650	1,548	1,098
Muhazi/Rwamagana	2,885	495	700	495	2,390
Ryarubamba/Gatsibo	400	100	0	800	0
Total	5,931	1,493	1,350	2,843	3,488
